# Oxidative Priority, Meal Frequency, and the Energy Economy of Food and Activity: Implications for Longevity, Obesity, and Cardiometabolic Disease

**DOI:** 10.1089/met.2016.0108

**Published:** 2017-02-01

**Authors:** Raymond J. Cronise, David A. Sinclair, Andrew A. Bremer

**Affiliations:** ^1^ Thermogenex, Huntsville, Alabama.; ^2^ Department of Genetics, Harvard Medical School, Boston, Massachusetts.; ^3^ Department of Pharmacology, School of Medical Sciences, The University of New South Wales, Sydney, Australia.; ^4^ Division of Diabetes, Endocrinology, and Metabolic Diseases, National Institute of Diabetes and Digestive and Kidney Diseases, National Institutes of Health, Bethesda, Maryland.

**Keywords:** oxidative priority, metabolism, obesity, longevity, cardiometabolic disease

## Abstract

In most modern societies, the relationship that many individuals have with food has fundamentally changed from previous generations. People have shifted away from viewing food as primarily sustenance, and rather now seek out foods based on pure palatability or specific nutrition. However, it is far from clear what optimal nutrition is for the general population or specific individuals. We previously described the Food Triangle as a way to organize food based on an increasing energy density paradigm, and now expand on this model to predict the impact of oxidative priority and both nutrient and fiber density in relation to caloric load. When combined with meal frequency, integrated energy expenditure, macronutrient oxidative priority, and fuel partitioning expressed by the respiratory quotient, our model also offers a novel explanation for chronic overnutrition and the cause of excess body fat accumulation. Herein, we not only review how metabolism is a dynamic process subject to many regulators that mediate the fate of ingested calories but also discuss how the Food Triangle predicts the oxidative priority of ingested foods and provides a conceptual paradigm for healthy eating supported by health and longevity research.

## Introduction

In just over two centuries, we have advanced from discovering the role of oxygen in respiration to interrogating a complex, molecular biology-based model of cellular respiration, hormonal regulation, and bioenergetics in human physiology and pathophysiology. Over a century ago, food was systematically analyzed for its basic energy components—protein, carbohydrate, and fat—and the bioavailability of this energy quantified as a basis for nutritive economic exchange. Direct and indirect calorimetry experiments on individuals in both long- and short-term respiration chamber studies involving both eating and physical activity have also thoroughly documented energy conservation. Why, then, do popular self-help books, scholarly articles, and media headlines still question the validity of the dietary calorie (technically a kilocalorie [1000 cal] or ∼4.2 kJ) in predicting energy deficit and accumulated excess? And why, despite more being known about energy management than ever before, does our society appear incapable of managing an otherwise simple energy balance?

For many people in developed nations, their relationship with food is fundamentally different than previous generations, with a shift away from sustenance and toward either pure palatability or specific nutrition (*i.e.*, high-protein diets, low-fat diets, low-carbohydrate diets, or combinations thereof). It is far from clear, however, what optimal nutrition is for the general population or for specific individuals. And, how does one make sense of all the claims of certain dietary patterns in addition to the ubiquitous conflicting advertising adjectives of fresh, organic, gluten free, low fat, low “carb,” or sugar free?

As we previously described,^1^ the Food Triangle represents a new organization of food based on an increasing energy density paradigm. However, the Food Triangle can further be expanded to predict the impact of oxidative priority and both nutrient and fiber density in relation to caloric load. When combined with meal frequency, integrated energy expenditure, and fuel partitioning expressed by the respiratory quotient (RQ), our model also offers a novel explanation for chronic overnutrition and the cause of excess body fat accumulation.

In this short review, we will discuss four main points: (i) nutrition is not an emergency; (ii) the Food Triangle predicts oxidative priority; (iii) the chronically fed state is the antithesis of dietary restriction (DR) and counters the favorable metabolic effects of DR demonstrated in longevity research; and (iv) exercise-induced shifts in RQ disrupt fat disposal and portend an unintentional cascade of feeding events.

## Calorie In, Calorie Out

One of the original tenants of human metabolism is that ingested food can be quantified as energy exchange units based on biological energetics and associated waste heat. Throughout the second half of the 19th century and an early part of the 20th century, researchers worked carefully to define this exchange equivalency, or digestive efficiency.^2–6^ Ingested food is broken down into components of energy that are stored, burned, or eliminated in waste. By careful comparison of bomb calorimeter data for various foods with measurements of heat and respiration components in the hours following meals, Carl von Voit, Max Rubner, Max Pettenkofer, Wilbur Atwater, Francis Benedict, and others calculated the average energy conversions for protein, carbohydrate, fat, and alcohol.^5–10^ While it may be true that isocaloric foods are not always *isometabolic*, food caloric intake minus heat dissipated, respiration, and those calories eliminated as fecal and urine waste must equal zero.

While it may appear that certain calories dominate fat loss and gain, the discrepancy is generally attributed to an oversimplification of energy accounting. With an exception of rare genetic disorders, obesity is largely caused by excessive food intake, with a lesser contribution from physical inactivity due to the tendency to increase calorie consumption during exercise.^4,11^ However, the phenomenon of an *acquired appetite* that dominates instinctual eating cues has been recognized since the time of Hippocrates^12^ and the mechanisms involved in leading some people to overeat while leading others to reach a natural balance between hunger and weight remain unresolved.^13,14^ Obesity is not common in the animal kingdom, with the exception of animals (*e.g.*, pets) we overfeed or when excess calories are unnaturally readily available. Moreover, in a calorically scarce environment, a desire to overconsume is not a trait of negative selection and may in fact assert positive benefits. As we describe below, the tendency to store the most energy dense portion of a meal (*i.e.*, fat) may also be explained in terms of an adaptive advantage.

## Thermodynamics of Overnutrition

**FIG. 1.**
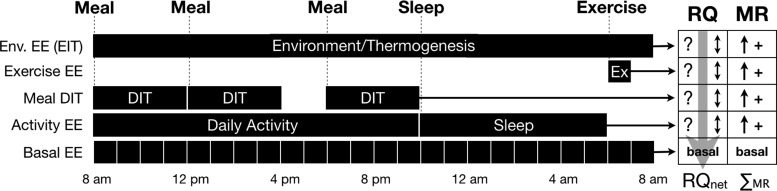
BMR modification and associated impact on fat oxidation. For each 1-hr interval during a 24-hr period of time, both the MR and RQ must be evaluated to accurately calculate net loss/gain of a specific substrate (*e.g.*, fat). While feeding, activity, and environment may all increase BMR, RQ is dynamic (not discrete for each activity) and acts on the instantaneous *total* MR. If the largest MR periods occur during times of highest RQ, stored fat is preserved. Use of an average or predicted metabolic rate over a 24-hr period of time compared to calories actually ingested is insufficient to accurately predict substrate gain/loss. BMR, basal metabolic rate; DIT, dietary-induced thermogenesis; EE, energy expenditure; MR, metabolic rate; RQ, respiratory quotient.

While it is arithmetically convenient to focus on a daily calorie surplus or deficit, the specific substrate or nutrient utilized, along with energy increases from basal requirements due to diet, activity, and the environment, must be examined on a shorter time scale for a more accurate representation of fatty acid accumulation or oxidation. Figure 1 illustrates calorie input and disposal for a typical day using 1-hr time intervals with the indicated duration, frequency, and overlap of primary energy components. Daily total energy expenditure (TEE) is a composite of basal metabolic rate (BMR), activity, exercise, dietary-induced thermogenesis (DIT), and environmentally-induced thermogenesis (EIT). Experimentally, BMR is measured in the fasted state upon waking in a dark room. However, BMR is often used synonymously with resting metabolic rate (RMR), which typically is measured less stringently and requires only fasting and the absence of physical activity, typically 4 hr before the test.^15^ Experimental tests for both BMR and RMR are normally 15–20 min in duration, and the results extrapolated over a 24-hr period of time.^15^

Early calorimeter experiments carefully measured the heat emitted from a thermally isolated room yielding *direct calorimetry* measurements over hours and even days.^16^ Later, in part, for practical purposes, most researchers turned to measuring carbon dioxide (CO_2_) produced and oxygen (O_2_) consumed to give an *indirect calorimetry* measurement of the heat evolved from respiration. The first systematic investigation of the gross energy content of food is credited to Rubner in Germany and Atwater in the United States.^7^ By comparing indirect calorimetry of subjects with a growing database of direct bomb calorimetry of more than 4000 analyses of 1360 different food items, Atwater improved on Rubner's earlier work and created a range of factors and coefficients representing energy availability and digestibility of foodstuffs to delineate their useful calorie content.^17^ However, the oversimplified and generalized notion of energy storage being a consequence of “calories in and calories out,” often interpreted as diet and exercise, fails to capture the more subtle thermodynamics of energy storage and disposal, particularly waste heat. Moreover, the human body is not a perfect engine and it is known that the energy liberated from the *combustion* of food is not identical to the energy available to the body from the *consumption* of food.^18^ This concept, known as “metabolizable energy,” is the difference between the gross energy of consumed food measured by bomb calorimetry and the energy contained in the feces and urine.^19^

**FIG. 2.**
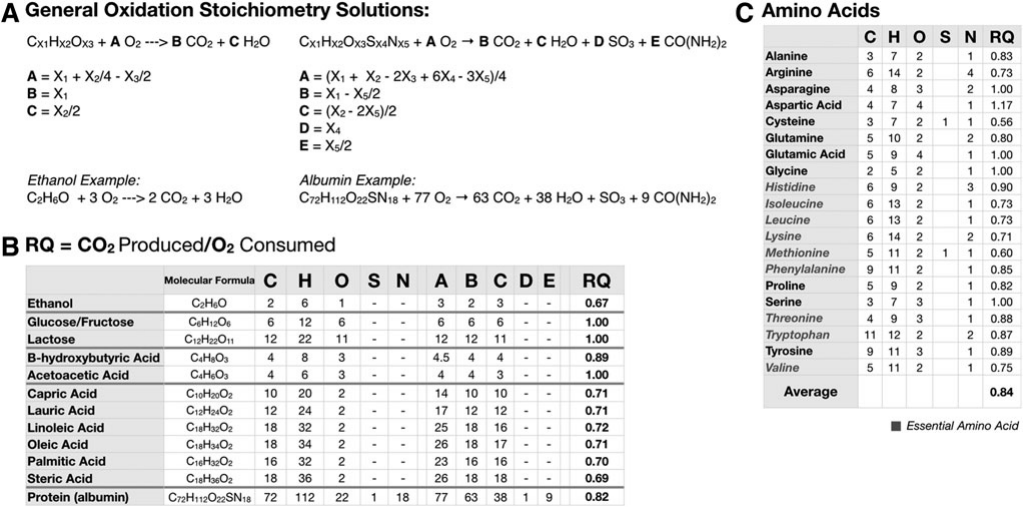
Substrate oxidation and RQ. **(A)** General stoichiometry solutions for various fuel substrates. **(B)** The cellular RQ—the ratio of CO_2_ produced to O_2_ consumed—for a wide variety of foodstuffs. **(C)** Individual amino acid RQ values are summarized; the average RQ for all amino acids is an RQ = 0.84. Generally accepted RQ values for the major macronutrients are protein (PRO) = 0.82; carbohydrate (CHO) = 1.0; and fat (FAT) = 0.70.

To address the dynamic nature of metabolism, the RQ must be factored into this composite metabolic summary. RQ can be estimated by the respiratory exchange ratio (RER), or the ratio of CO_2_ produced and O_2_ consumed during respiration, and can be directly related to the individual substrate being oxidized, whether it is protein, carbohydrate, fat, or alcohol.^20^ A general stoichiometric solution for protein, carbohydrate, fat, alcohol, and other less abundant carbon sources in the diet such as acetate and ketone bodies is shown in Fig. 2. The significance of the RER measurement is the ability to use it to estimate the RQ of a mixture of fuel sources (*i.e.*, a typical meal of mixed macronutrients).^15^ Generally, lipids have an RQ of ∼0.7 and carbohydrates have an RQ of ∼1.0. The RQ of alcohol is slightly lower than lipid at ∼0.67, and while the RQ of amino acids range from 0.6 to 1.17, the average is ∼0.84. In the case of proteins, since amino acids are not completely oxidized, urine and fecal urea is collected over a 24-hr period to calculate protein oxidation; this value is then subtracted from RQ measurements to give nonprotein RQ (npRQ).^15^ Fortuitously, because the RQ values of proteins generally fall in the middle of the carbohydrate/fat range, indirect calorimetry gives a relatively accurate quantitative snapshot of carbohydrate/fat energy partitioning without nitrogen balance.^15^ For more complete and extensive assessments of energy partitioning, the npRQ is used.

For an accurate interpretation of energy homeostasis, the importance of RQ cannot be overemphasized. One must consider not only the quantity of each ingested macronutrient but also, more importantly, how these fuels are stored and utilized. A surplus or deficit of fat cannot be calculated if one only considers a 24-hr extrapolation (TEE) of a 15-min measurement (BMR/RMR) and compares the resulting total predicted activity output with actual calories consumed unless it is also accurately known how the macronutrients are partitioned as fuel throughout the day.

Importantly, RQ captures the percentage rise and fall of fat utilization driven by diet, activity, and environment. And, by combining RQ with TEE, it is evident that the utilization and storage of fat are most relevant to weight gained or lost; this can be represented mathematically as follows:

\begin{align*}
\left[ { \int \limits_0^{24} { ( { \rm{FAT \% }} ) { \rm{EE}}dt} } \right] - { \rm{FAT}} \ { \rm{INTAK}}{{ \rm{E}}_{24 \ { \rm{hr}}}} = { \rm{FAT}} \ { \rm{Loss / Gain}}
\end{align*}

Despite numerous studies demonstrating that *de novo* lipogenesis from carbohydrates does not significantly contribute to the increase in whole-body adipose tissue stores found in obesity,^21–27^ the notion that “sugar turns to fat” abounds. Perhaps there is further confusion resulting from the unintented consequences of aggregating whole-food complex carbohydrates with highly refined grains and sugars simply because they share the defining glycosidic bond found in all carbohydrates. Not only does this detract from the important negative metabolic effects that excess simple sugar consumption has on hepatic steatosis and mitochondrial dysfunction but it also obfuscates the role that excess dietary protein (amino acid), alcohol, and carbohydrates play in driving dietary fat storage and interfering in stored adipose disposal through normal metabolic activities during the fasted state.

So, how can generalized notions of metabolism be reframed to provide a more accurate assessment of fat disposal and accumulation? Consider the fasted and inactive BMR during a 24-hr period as shown in Fig. 1. During each hour of fasting, the fat deficit grows and its magnitude is determined by the fat percentage of TEE in each unit time. Upon waking and with nondepleted glycogen stores, a normal fasted RQ is ∼0.85, or ∼50% oxidation both from stored fat and carbohydrate. In this scenario, about half of the expected calorie deficit for the 24-hr TEE is predicted to be from fat. Next, consider added activity and its associated increase in energy expenditure (EE) during the active period along with associated increases in RQ (favoring carbohydrate oxidation) typical with periods of increased physical activity. Note that for each hour of activity-shifted RQ and increased EE, the “new” RQ not only affects the calories expended by the added activity but also impacts the calories that *would* have been expended by the BMR for that same period. This creates a seemingly paradoxical situation, as it is possible to significantly *increase* EE with activity and simultaneously *decrease* fat percentage utilization to a point of diminishing returns when compared to calorie restriction alone.

**FIG. 3.**
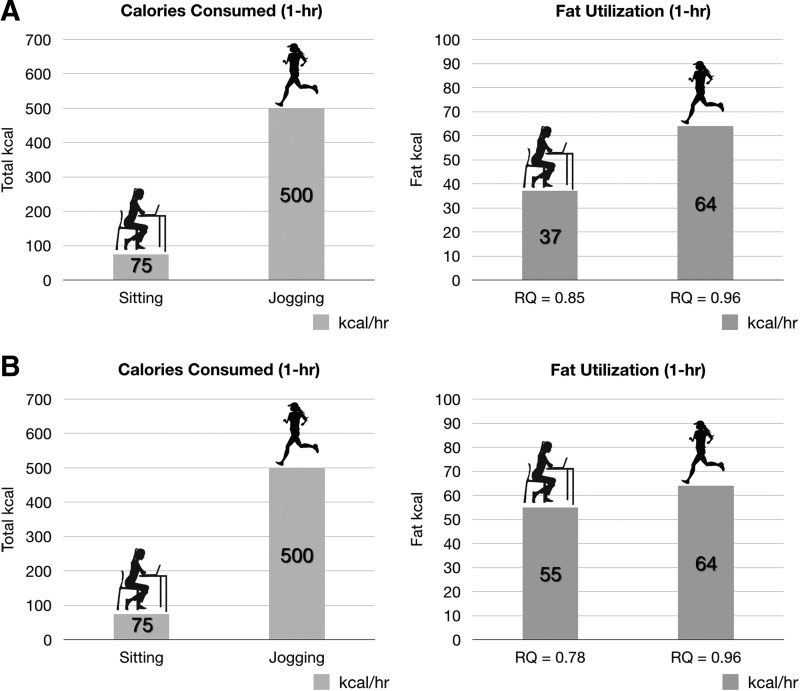
Fuel utilization and partitioning with physical activity versus calorie restriction. A 1-hr block of time spent sitting is compared to a 1-hr block of time spent jogging. **(A)** At an RMR of 1800 kcal/day (a typical individual), a predicted resting RQ of 0.85 (49.3% fat oxidation) results in 75 kcal/hr EE and 37 kcal/hr fat utilization. During activity, the rise in RQ to 0.96 (12.8% fat oxidation) results in 500 kcal/hr EE and 64 kcal/hr fat utilization. For comparison, the change in fat utilization between rest and activity equates to slightly more than a ½ teaspoon of olive oil (∼40 kcal/tsp). **(B)** During times of sustained calorie restriction, resting RQ may drop to an RQ of 0.78 (∼74% fat oxidation) due to increased fat utilization, resulting in ∼55 kcal/hr fat utilization. This is a difference of only 9 kcal (less than a ¼ teaspoon of olive oil) from the 64 kcal/hr fat utilization predicted during physical activity in a noncalorie-restricted state. EE, energy expenditure; RMR, resting metabolic rate.

For example, as illustrated in Fig. 3, assume the BMR for a person is 1800 kcal/day (75 kcal/hr). One hour of physical activity (*i.e.*, jogging at 5 miles/hr) will result in an increase to ∼500 kcal/hr in EE. However, it is the utilization of fat calories (resulting from shifts in RQ in any given time unit) that is more relevant and important than total EE when it comes to body weight. As demonstrated in many calorimeter experiments, vigorous activity will tend to raise RQ and therefore decrease percent fat utilization during the activity period.^9,28,29^ Alternatively, an individual in the fasted/resting state or during extended caloric restriction will have a decreased RQ, thereby increasing percent fat utilization.^30,31^ Conceptually it might be difficult to equate 1 hr of jogging with a little more than half a teaspoon of olive oil (∼40 kcal/tsp) with respect to energy equivalents. However, this observation readily explains why any increase in EE induced by exercise can quickly be overcome by diet. While it is often argued that exercise preserves fat-free mass, when calorie restriction and calorie intake plus exercise are precisely matched, there seems to be parity in loss.^11^ Furthermore, Redman et al. suggest that individuals are genetically or epigenetically programmed for fat loss and accumulation, a “first on-last off” response that may explain why problem areas, such as abdominal fat, are variable among individuals of similar fitness.^11^ Finlayson et al. also suggest that the benefits of exercise may be offset in some people by an “individual reward,” in which an increased desire to eat compensates for any calories lost during the activity.^32^ We propose that determining the exact cause of weight change may be additionally masked by thermodynamic shifts in fuel partitioning through a complex interaction of activity, calorie source, and environment.

## Thermodynamics of Feeding

**FIG. 4.**
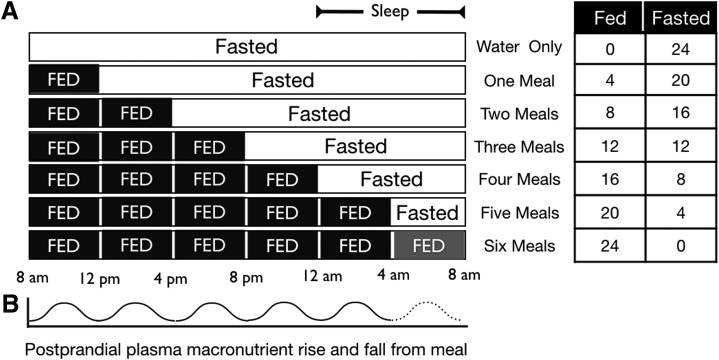
Fed and fasted state versus meal frequency. **(A)** Different meal frequencies and their effects on the fed/fasted state. **(B)** Postprandial fluctuations in plasma macronutrient levels based on meal frequencies.

Feeding, with associated DIT and oxidative priority, adds another level of complexity to the “is a calorie a calorie?” question. Figure 4 illustrates the impact of meal frequency on the time available to dispose of ingested energy. In the fasted state, metabolic energy is completely sourced from body stores. If the fast continues, glycogen stores will eventually be depleted and a shift to fat oxidation will ensue.^33^ Fatty acid-derived ketones can not only contribute to metabolic activity but also provide up to 60%–70% of brain energy needs during long periods of glycogen depletion; blood glucose concentrations are maintained by gluconeogenesis and glucogenic amino acids sourced from protein stores.^34,35^

On feeding, a complex set of digestive processes are initiated, which result in the breakdown of the ingested foodstuffs into their constituent macronutrients (and micronutrients) and their distribution to the tissues. Importantly, by considering the fed and fasted state in a more general conceptual model, the problem that may unfold through chronic overnutrition becomes evident. As each successive meal is added, it disrupts the fasted state and obligates the body to take action on the newly ingested fuel. And although each meal is associated with a 4–6-hr postprandial increase in metabolic rate,^36^ the fate of the ingested fuel is discrete as it will be either metabolized, stored, eliminated, or a combination thereof. All calories ingested must ultimately meet one of these three ends. Since meals consist of a mixture of macronutrients, the logical question centers on the regulation of fuel partitioning after a meal: how does the body decide which fuel to utilize, eliminate, or store?

## DIT and Oxidative Priority

**FIG. 5.**
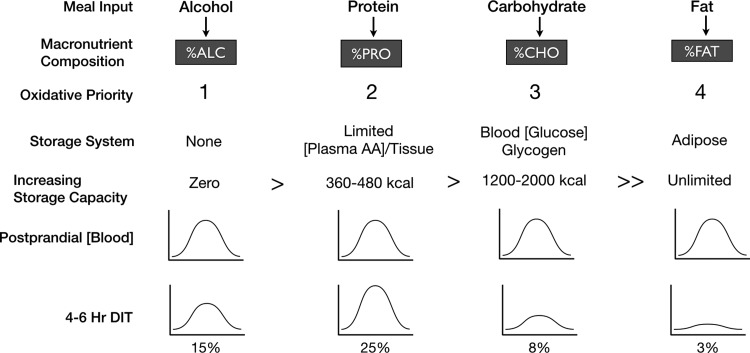
Oxidative priority of macronutrient disposal. An oxidative prioritization of macronutrients predicts the partitioning hierarchy based on the inverse relationship of storage capacity. Not all macronutrients can be stored at the same level. For example, alcohol has no long-term storage capacity, while fat has a relatively unlimited capacity. While the postprandial DIT rise is highest for protein and lowest for fat, these metabolic increases do not necessarily reflect utilization of stored energy reserves and are rather better explained by the need to mitigate postprandial rises in blood nutrient levels. In nearly all cases, postprandial blood analyte levels that increase due to meals normalize within 4–6 hr as the postabsorptive state is reached. ALC, alcohol; CHO, carbohydrate; DIT, diet-induced thermogenesis; FAT, fat; PRO, protein

Prentice and colleagues addressed the issue of fuel partitioning with their model of oxidative priority, or *oxidative hierarchy*, based on individual macronutrient storage (Fig. 5).^37–44^ Conceptually, to better understand the fate of the molecular components of food as they begin to leave the digestive tract, it may be useful to put aside the popular food labeling and macronutrient organization scheme of protein, carbohydrate, and fat, and recognize that whole foods, especially entire meals, are mixtures. Complex carbohydrates and simple sugars are broken down to monosaccharides, proteins into constituent amino acids, and lipids mobilized through chylomicron transport. Eating thus results in a postprandial rise in monosaccharide (glucose), amino acid, and lipid blood levels, during which the body attempts to normalize their concentrations by one of the three strategies: utilization, storage, or elimination. Importantly, when storage is limited or impossible, then two choices remain: utilize or eliminate.

For example, following the ingestion of a protein-rich meal, a relatively larger increase in DIT is observed in the postprandial state compared to a carbohydrate-rich meal since amino acids have no significant inherent storage depot; the body essentially transforms excess energy into waste heat. Although the postprandial rise in metabolism following a protein-rich meal may be considered advantageous and a way to mitigate weight gain or even lose weight, little if any of this excess energy is sourced from stored adipose tissue. As demonstrated with the increase in metabolic rate with physical activity, it is possible and even expected that increases in metabolism to dispose of excess calories ingested with a protein-rich meal may actually cause a decrease in stored adipose tissue utilization. An equally important consideration is the following: not only may dietary fat be stored at a higher rate in the presence of macronutrients with a higher oxidative priority but also overall fat oxidation rates may be decreased by shifts in fuel partitioning.^45^

Conversely, a greater thermogenic response to food may *not* always cause excess stored energy reserves to be burned. This concept is demonstrated by data showing the effects of glucose and ethanol administration directly through intravenous infusion.^45^ Whereas ethanol and glucose infusions have no significant impact on overall RMR, ethanol alone suppresses fat oxidation by 87%. Importantly, a glucose infusion following ethanol predictably increases carbohydrate oxidation by 249% and further suppresses fat oxidation to almost nil for 90 min, with only a slow rise in fat oxidation thereafter; in fact, the total 4-hr integrated oxidation of fat following an ethanol-then-glucose infusion is suppressed by 79%.

Returning to the concept of meal frequency and mixed-meal oxidative priority, one can translate these results to what occurs when an individual consumes a wine, cheese, and cracker hors d'oeuvre. The post-hors d'oeuvre rise in alcohol and glucose concentrations actually suppresses fat oxidation and promotes storage of the dietary fat from the meal. As such, one can easily understand how body fat increases over time; the homeostatic drive to normalize postprandial rises in blood nutrient levels offers a novel conceptual framework that predicts fat accumulation.

## Adaptive Thermogenesis

An additional element that influences the body's metabolism is the overall environment—particularly mild cold stress.^46–49^ In contrast to physical activity that increases RQ, the body's environment can induce the opposite effect.^29,50^ For example, long periods of mild cold stress from cooler ambient temperatures can induce nonshivering thermogenesis, reducing RQ (favoring fat oxidation)^51^; this, simultaneously, increases fasted/resting EE and may lead to an overall increased fat deficit.

Specifically, the activation of brown adipose tissue (BAT) increases mitochondrial heat production in skeletal muscle cells,^50,52–54^ at least, in part, by the upregulation of peroxisome proliferator-activated receptor-γ coactivator (PGC)-1α—which is induced by low caloric intake or mild cold stress.^52,53,55–58^ Why PGC-1α is upregulated in response to both low caloric intake and mild cold stress is yet to be fully elucidated; but, in our “Metabolic Winter Hypothesis,”^1^ we postulate that the molecular responses to these two stressors (*i.e.*, caloric restriction and cold temperatures) may have evolved in tandem—both conserving energy in the fasted state and partitioning calories to fat for warmth during times of cold. Furthermore, as reported by both Valdés et al.^59^ and Yang et al.^60^ studying relatively homogeneous genetic populations consuming a comparable diet living at mildly different degrees of latitude, an association exists between ambient temperature and obesity.

In general, a discussion of “calories in and calories out” often includes generalized metabolic rates, DIT, energy associated with physical activity, and even BAT and mild cold stress. However, ultimately, it is the *integrated* total energy expended and fuel partitioned (metabolic rate and RQ) that determine how much adipose tissue is depleted in a given 24-hr period. During periods of nonglycogen depleted fasting, this might be a 50:50 carbohydrate:fat split (RQ ∼0.85). In the postprandial/fed state, fat utilization may be significantly reduced; alternatively, during an extended fast (glycogen-depleted ketosis), fat utilization may dominate. Clearly, the simple average metabolic rate summary and ubiquitous focus on metabolic “boosting” diets and exercise are insufficient to explain the thermodynamics of feeding; importantly, they may have an unintended consequence in metabolic rate modification and unanticipated RQ shifts away from fat utilization.

## The Food Triangle and Whole-Food Diet Schemes

As we explained in more detail previously,^1^ the Food Triangle offers a novel way to conceptualize food in a manner that eliminates the traditional macronutrient food groupings of protein, carbohydrate, and fat that are in widespread use. The Food Triangle organizes whole food using an energy density paradigm. It recognizes that essential amino acids (*i.e.*, proteins) are not limiting nutrients in any whole-food diet that meets daily energy needs. Rather, vegetable- or animal-sourced amino acids, in excess of daily requirements, along with carbohydrates and fat, all become fuel with oxidative priority given to that which is not readily stored. This organization of energy density permits individuals to address their micronutrient requirements (the apex of the triangle) without driving chronic overnutrition (the bottom vertices of the triangle). These foods become the nutritional foundation of daily meals, rather than the more energy-dense alternatives. They also provide a rich source of phytonutrients and can be eaten in nearly unlimited quantities. It further places emphasis on foods that are increasingly important for healthy gut microbiota.

**FIG. 6.**
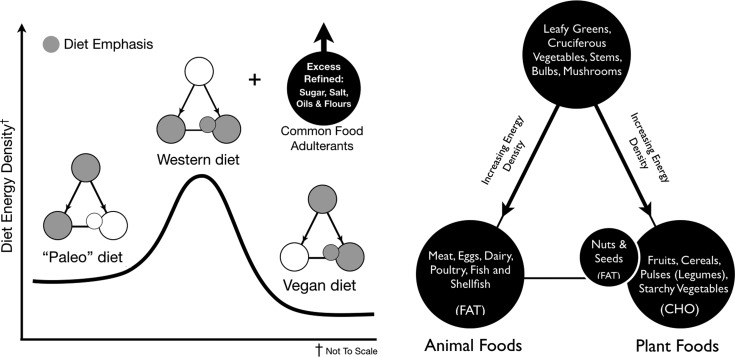
Energy density of popular diet schemes. Our version of the Food Triangle may be used to compare popular diet schemes for energy density. For example, the relative energy density of a Paleo- versus vegan-style diet, which nearly mirrors the opposite sides of the Food Triangle, is shown. Furthermore, a “Western diet,” which is more heavily weighted toward foods at the base of the Food Triangle, is predicted to result in maximum energy accumulation of dietary fat. While not considered part of the whole foods integrated into the Food Triangle, excess refined oil, sugar, and grain (*e.g.*, flour) adulterants may drive any of these diet schemes to unintentional chronic overnutrition. While either side of the Food Triangle is predictive for excess energy, it may also be used to predict overnutrition of dietary amino acids and fats and undernutrition of phytonutrients and dietary fiber.

By utilizing the Food Triangle and combining the effect of fed/fasted periods, dietary- and activity-induced RQ shifts, and oxidative priority, it is possible to predict substrate utilization and how different diet schemes effectively mediate preferential fat disposal (Fig. 6). While the magnitude of the impact of carbohydrate consumption versus fat consumption on fat mass gain has been deliberated for decades, the Food Triangle provides a conceptual framework of the relationship between diet and energy density and suggests that both (*i.e.*, dietary carbohydrates [sugars] and dietary fats) individually and synergistically contribute to fat mass gain. We recognize that the complex interdependent roles of insulin, glucagon, and incretins^61,62^—in addition to other hormones and regulatory molecules—in both the fasting and postprandial states provide mechanistic insights into *how* oxidative priority is managed and are not comprehensively addressed in this review.

However, our objective with this essay is not to provide an extensive review of nutritional biochemistry and endocrinology; rather, it is to (1) focus on *why* social influence and unnatural access to food have created the opportunity for the chronic postprandial state and (2) organize food in an understandable manner that much more readily predicts fat accumulation. This conceptual framework also explains why diets that eliminate one or the other (*i.e.*, diets that eliminate fats but permit carbohydrates or vice versa) might be effective in the short term, but are typically unsuccessful for sustained weight loss when the eliminated food is returned. The apex of the Food Triangle contains phytonutrient/fiber-rich, low-calorie foods. Down each descending side of the Food Triangle are foods of increasing energy density separated as primarily fat- and carbohydrate-sourced food. A notable exception is the fats sourced from whole-food plant-based nuts, seeds, and certain fruits (*e.g.*, avocados and coconuts).

If one eats on the right side of the Food Triangle, primarily sourcing energy from whole-food dietary starches and fruits, dietary carbohydrate (beyond that needed to replete glycogen stores) may be disposed of via increased RQ and postprandial EE via DIT. On the contrary, a diet sourced from the left side of the Food Triangle will lead to depleted glycogen stores and eventually induce a ketogenic state, lowering RQ and favoring fat utilization. Unlike excess dietary carbohydrates, which are disposed of through oxidation, excess dietary lipid may be partially eliminated in the feces after incomplete absorption. Each approach may be effective in inducing the calorie deficit required to result in weight loss; but, long-term lifestyle decisions to increase an individual's healthspan are more complex. The Food Triangle thus fundamentally provides a paradigm of healthy eating that extends beyond the caloric component of foodstuffs.

Diets that emphasize the base of the Food Triangle—perhaps even as an unintended consequence of what one might call a “balanced meal”—may drive excess fat storage via the mechanisms of oxidative priority described above. And, as shown in Fig. 6, the oxidative priority model portends that the postprandial partitioning and disposal of foodstuffs from a typical “Western” diet (*i.e.*, a diet emphasizing the base of the Food Triangle) would be obesogenic; this prediction matches the current reality.^63^ Bottom feeding on the base of the Food Triangle, especially with the added consumption of refined grains (*e.g.*, flours), oil, and sugar adulterants, yields a diet in which the most energy-dense foods are consumed together. Many traditional meal plans from a century or more ago aimed to mitigate economic calorie scarcity, but may have unintentionally created today's widespread food culture-induced bottom feeding (*e.g.*, burger-fries, steak-potatoes, fish-chips, pasta-meat sauce, curry-rice, etc). However, in an environment of unnatural calorie availability, these meals become maladaptive.

This, combined with predictive oxidative priority and a regulated capacity to store excess carbohydrates as glycogen, favors fat storage for later use; unfortunately for most, later never comes. Modern successes in agriculture, reduced food cost, and availability—coupled with current sleep patterns—enable a chronically fed state; and, with excessive meal/snack frequency, it is possible for many individuals to remain in the postprandial/absorptive state for most of the day. This is a period when ingested calorie disposal, not the utilization of energy reserves, is prioritized.

The chronically fed state is further encouraged by the common notion that “nutrition is an emergency” and that frequent feeding is beneficial, despite numerous studies suggesting the beneficial effects of intermittent fasting, diets that mimic fasting, calorie restriction, protein restriction, and prolonged medically supervised water fasts.^64^ However, not only do calorie restriction, alternate day fasting, and periodic fasting have a long history in society, they are the only mechanisms by which life/healthspan has been extended in the laboratory.^65–70^

Researchers and dietitians alike also continue to focus on the “eat meat versus do not eat meat” or “fat versus sugar” debate. We propose this constitutes a false ideological dichotomy that only loosely addresses fat accumulation over time. For example, if one were to follow a strict vegan diet based on the right side of the Food Triangle, thereby eliminating all dietary fat sourced from animal products without limiting oils, refined grains (*e.g.*, flours), or sugar, it is still possible to drive dietary fat storage. Likewise, diets on the left side of the Food Triangle can drive dietary fat storage despite the oft-cited health benefits of maintaining a state of relative ketosis. Why is this so? Because it does not take much dietary carbohydrate, even from healthful sources such as fruits and whole-food low-glycemic starches, to eliminate ketosis and increase the postprandial RQ to favor carbohydrate disposal. In this case, although the dietary carbohydrate is typically viewed as the culprit for fat storage, it is actually the dietary fat component of the meal that is stored. Hall et al. have also reported that consuming an isocaloric low-carbohydrate ketogenic diet when compared to a high-carbohydrate baseline diet is not accompanied by increased body fat loss despite a relatively small increase in EE, or metabolic advantage.^71^

This creates a dietary stalemate; and, while energy components are only one dimension of the Food Triangle, they clearly predict the accumulation of excess adipose tissue when combined with the conceptual model of oxidative priority and hours spent in the fed versus fasted state. The two primary dietary fuels, fats and carbohydrates, essentially exert competing excess energy challenges: dietary carbohydrate calories displace stored fat energy that would have been utilized in the fasted state and dietary fat accumulates, increasing the fat burden that must be used in the future. Clearly, in the chronically fed state, these two energy imbalances favor net accumulation of adipose tissue and in the calorie-limited world this would be an adaptive evolutionary advantage for infrequent feeding opportunities. However, most of the inhabitants of developed countries do not live in a calorie-limited world. Another benefit of returning the discussion back to whole foods using the Food Triangle rather than grouping dietary components in overly general categories of protein, carbohydrates, and fats is that one needs more specificity on each of these macronutrients (*e.g.*, essential vs. nonessential amino acids, simple monosaccharides versus complex carbohydrates and dietary fiber, and saturated and trans-unsaturated fatty acids versus mono- and polyunsaturated fatty acids) to study and assess their impacts on health.

## Implications for Cardiometabolic Disease

The increased prevalence of overweight and obesity over the past several decades has paralleled the increased prevalence of cardiometabolic disease.^69,72,73^ Although the correlation between excess weight and cardiometabolic disease is not sufficient to demonstrate causality, most researchers agree the two are tightly linked. Moreover, weight loss via lifestyle factors such as diet and exercise remains a cornerstone to therapy for cardiometabolic disease. However, the *relative* contribution of diet and exercise to weight loss remains controversial.^74,75^

Furthermore, while potential exercise-induced shifts in eating habits and RQ may lead to less fat utilization in the short term, they should not overshadow the myriad health benefits of exercise or discourage physical activity. However, the option to delay or suspend excessive physical activity during a period of decreased nutrient availability (*e.g.*, during a period of dietary caloric restriction) is a viable alternative to increasing nutrient intake to meet the increased metabolic demands of exercise. Decreased nutrition combined with increased physical activity may unintentionally limit recovery; and, depending on a person's frame, excessive fat mass may increase the risk of injury. However, these issues are mitigated as one approaches normal weight.

From a conceptual framework, it is also important to recognize two important points: (1) although both caloric restriction and exercise may lead to weight loss, the physiological adaptation and responses to each differ dramatically and (2) the weight loss-independent effects of caloric restriction and exercise on risk factors for cardiovascular disease are not additive.^76^ However, because physical inactivity and poor aerobic capacity are independent risk factors for cardiovascular disease, exercise—when safely performed at an appropriate weight—may provide a benefit that cannot be achieved by caloric restriction alone.^76^

As reviewed above, the thermodynamic responses to ingested foodstuffs clearly differ from physical activity. Although many individuals consider exercise to be an antidote to excess calorie intake and that physical activity without changes in dietary intake may lead to significant weight loss, the available data suggest otherwise.^11,77–79^ In general, the negative impact of modern diets exceeds the positive impact of exercise on net weight. And, although the factors that lead to obesity and the components of the metabolic syndrome that often (but not necessarily) accompany obesity are not identical, they are nonetheless associated with cardiometabolic disease risk.^80–82^

## Conclusion

From an evolutionary adaptive perspective, it seems unlikely that our ancestors had such easy access to the current socially normal and excessive meal frequency and calorie density. Although periods of fasting for days or perhaps even weeks may appear socially extreme today, they are tolerable^83–88^ and were likely imperative to survive in an environment that historically humans had little to no control over. Moreover, seasonal and circadian zeitgebers exert strong feedback mechanisms on cellular and whole-body function, including effects on nutrient disposal.^89–95^ Our “Metabolic Winter Hypothesis” suggests that a healthful existence requires the majority of us to reassess our relationships with food intake, sleep, and the temperature of our environment.^1^ Many observations indicate that DR, mild cold stress, and sleep all appear to have a related mechanistic role of increasing health and longevity and mitigating age-related diseases.^96,97^ One may conceptually frame this by contrasting a metabolic winter (*i.e.*, cool, dark, still, and scarce) with a metabolic summer (*i.e.*, warm, bright, active, and abundant). Added to this framework are the myriad cell signaling pathways and fundamental cellular processes modulated by nutrient availability and nutrient signaling. These are likely part of a larger phenology influencing all animal and plant life, a phenomenon that humans have successfully engineered out of their daily life.

Importantly, using the Food Triangle in place of the traditional categorization of foods as protein, carbohydrate, or fat allows one to more easily identify eating patterns that will likely lead to weight gain. Moreover, food stuffs on the right side of the Food Triangle have components that longevity research shows can extend the healthspan, if not the life span, of mammals and potentially humans^98^ by minimizing essential amino acids to decrease mTOR and GH/IGF-I signaling, while maximizing dietary fiber, vitamins, minerals, and micronutrients such as carotenoids, phytosterols, and flavonoids to activate sirtuins and AMPK.^99–101^

If nutrient availability plays a critical role in seasonal environmental signaling, it may raise the question of how various forms of DR impact overall fuel partitioning and disposal. For example, the restriction of methionine—found in high concentrations in animal-based protein diets (the left side of the Food Triangle) and low concentrations in plant-based protein diets (the right side of the Food Triangle)—has been associated with increased longevity and improved age-related health.^102–104^ One might also ask the question whether there is a difference in metabolic feedback to a whole food plant-based very low-calorie diet (VLCD) that has *restricted* nutrients versus the often abundantly supplemented modern VLCDs (*e.g.*, liquid diets). Furthermore, it is unclear from the standpoint of adaptive evolutionary pressure how any human ancestor might have experienced periods of severely scarce calories yet have had access to abundant “supplemental” nutrients.

In closing, over a century ago, Carl von Voit wrote in a memorial tribute to Max Pettenkofer after his death^7^:

Imagine our sensations as the picture of the remarkable processes of the metabolism unrolled before our eyes, and a mass of new facts became known to us! We found that in starvation protein and fat alone were burned, that during work more fat was burned, and that less fat was consumed during rest, especially during sleep; that the carnivorous dog could maintain himself on an exclusive protein diet, and if to such a protein diet fat were added, the fat was almost entirely deposited in the body; that carbohydrates, on the contrary, were burned no matter how much was given, and that they, like the fat of the food, protected the body from fat loss, although more carbohydrates than fat had to be given to effect this purpose; that the metabolism in the body was not proportional to the combustibility of the substances outside the body, but that protein, which burns with difficulty outside, metabolizes with the greatest ease, then carbohydrates, while fat, which readily burns outside, is the most difficult to combust in the organism.

Indeed, metabolism is a dynamic process subject to many regulators that mediate the fate of ingested calories. Our version of the Food Triangle predicts the oxidative priority of ingested foods and provides a conceptual paradigm for healthy eating supported by health and longevity research. Importantly, the thermodynamics of feeding are also influenced by the fed and fasted states, and exercise-induced shifts in RQ differentially affect nutrient disposal. The typical modern existence in the chronically fed/postprandial state, combined with oxidative priority and the abundance of inexpensive, calorie-dense foods, and an aversion to mild cold stress amalgamate to predict weight gain for the majority of the developed world if current trends continue.
